# PLSCR1 suppresses SARS-CoV-2 infection by downregulating cell surface ACE2

**DOI:** 10.1128/jvi.02085-24

**Published:** 2025-02-13

**Authors:** Ruiyi Ma, Xinyi Zhang, Ruonan Li, Xiaojing Dong, Wenjing Wang, Qi Jiang, Xia Xiao, Yujin Shi, Lan Chen, Tian Zheng, Zichun Xiang, Lili Ren, Zhuo Zhou, Xiaobo Lei, Jianwei Wang

**Affiliations:** 1 NHC Key Laboratory of System Biology of Pathogens, and Christophe Merieux Laboratory National Institute of Pathogen Biology, Chinese Academy of Medical Sciences & Peking Union Medical College 12501 , Beijing, China; 2 Key Laboratory of Pathogen Infection Prevention and Control (Peking Union Medical College), Ministry of Education, Beijing, China; 3 Biomedical Pioneering Innovation Center, Peking-Tsinghua Center for Life Sciences, Genome Editing Research Center, State Key Laboratory of Protein and Plant Gene Research, School of Life Sciences, Peking University 117978 , Beijing, China; 4 Key Laboratory of Respiratory Disease Pathogenomics, Chinese Academy of Medical Sciences and Peking Union Medical College 12501 , Beijing, China; 5 State Key Laboratory of Common Mechanism Research for Major Diseases, Suzhou Institute of Systems Medicine, Chinese Academy of Medical Sciences & Peking Union Medical College, Suzhou, Jiangsu, China; University of North Carolina at Chapel Hill, Chapel Hill, North Carolina, USA

**Keywords:** SARS-CoV-2, PLSCR1, ACE2

## Abstract

Type I interferons exert their antiviral effects against SARS-CoV-2 by inducing the expression of interferon-stimulated genes (ISGs), including but not limited to LY6E, CH25H, IFITM2/3, and IFIH1. However, the antiviral effect and underlying mechanisms of action of most ISGs in SARS-CoV-2 infection are not yet fully understood. By screening 109 ISG-knockout cell lines, we identify that phospholipid scramblase 1 (PLSCR1), an interferon-inducible protein, acts as a crucial restriction factor against SARS-CoV-2 infection. Cells lacking PLSCR1 are highly susceptible to SARS-CoV-2 infection. Conversely, overexpression of PLSCR1 inhibits SARS-CoV-2 infection. Depletion of PLSCR1 enhances cellular entry of both pseudotyped and authentic SARS-CoV-2. Mechanistically, PLSCR1 inhibits SARS-CoV-2 entry by specifically downregulating plasma membrane expression of ACE2, the virus’s receptor, without affecting the overall levels of ACE2 within the cell. As such, we unraveled previously unappreciated mechanisms by which PLSCR1 exerts its restrictive effect on SARS-CoV-2. These data provide new insights into the interplay between host innate antiviral immunity and SARS-CoV-2 and shed light on novel antiviral therapeutics.

**IMPORTANCE:**

Phospholipid scramblase 1 (PLSCR1) has been identified as a critical host restriction factor against SARS-CoV-2 infection. In this study, we demonstrated that PLSCR1 inhibited SARS-CoV-2 entry by downregulating the plasma membrane expression of ACE2, the primary receptor for viral entry. Our findings elucidate a novel host-pathogen interaction that not only deepens our understanding of the innate immune response to SARS-CoV-2 but offers potential strategies for therapeutic interventions against COVID-19.

## INTRODUCTION

Since its emergence in December 2019, severe acute respiratory syndrome coronavirus 2 (SARS-CoV-2) has led to the novel coronavirus disease 19 (COVID-19) pandemic, which has posed a significant threat to global public health. The spectrum of symptoms associated with COVID-19 varies widely, ranging from self-limiting illness to severe or even life-threatening disease. The severity of COVID-19 can be influenced by various factors, including age, preexisting medical conditions, and immune response. The innate immunity system is the first line of defense against viral infection. Upon detection of viral invasion, infected cells and immune cells produce type I interferons, which play a crucial role in limiting viral infection. Growing evidence demonstrates that innate immune responses to SARS-CoV-2 infection can dictate clinical outcomes of COVID-19. Reportedly, at least 10% of patients with life-threatening COVID-19 pneumonia have autoantibodies that neutralize type I interferons (IFNs) (1). Additionally, 1%–5% of critical COVID-19 patients have genetic mutations that impair the type I IFN production or response to type I IFNs (2).

The binding of IFNs to their receptors activates the JAK-STAT signaling pathway, leading to the transcription of hundreds of ISGs. ISG-encoded proteins interfere with various stages of the viral life cycle, including viral entry, replication, and assembly. Several ISGs have been identified to specifically restrict cellular entry of SARS-CoV-2. For example, interferon-induced transmembrane protein 3 (IFITM3) (3, 4) and lymphocyte antigen 6 complex (LY6E) (5–7) block viral entry by preventing the fusion of the viral envelope with the host cell membrane. Cholesterol 25-hydroxycholesterol (CH25H), an enzyme, converts cholesterol into 25-hydroxycholesterol (25HC). Both the overexpression of CH25H and treatment of cells with 25HC have been shown to restrict SARS-CoV-2 infection, possibly by reducing cholesterol levels in both endosomal and plasma membranes, disrupting virus-cell membrane fusion (8–10). Nuclear receptor coactivator 7 (NCOA7) inhibits SARS-CoV-2 endocytic entry by interacting with vacuolar H-ATPase (V-ATPase), leading to abnormal vesicle acidification and lysosomal protease activation, thereby impeding SARS-CoV-2 entry (11). Phospholipid scramblase 1 (PLSCR1) has emerged as a host restriction factor against SARS-CoV-2 (12, 13). It inhibits SARS-CoV-2 entry by preventing spike protein-mediated fusion and viral entry (12).

SARS-CoV-2 utilizes two key proteins on the host cell surface for entry: angiotensin-converting enzyme2 (ACE2) and transmembrane protease serine 2 (TMPRSS2) (14, 15). Spike (S) protein is responsible for binding to the ACE2 receptor and facilitating viral entry. The entry process involves two main functional domains of the S protein: the receptor binding domain (RBD) in the S1 subunit that binds to ACE2, and the S2 subunit that mediates the fusion of the viral and host cell membranes. SARS-CoV-2 enters cells through two main pathways: plasma membrane fusion (early stage) and endosomal entry (late stage). The former pathway is more efficient and is triggered by TMPRSS2 present on the membrane surface. The virus can also enter cells by being endocytosed and then fusing with the endosomal membrane, a process activated by cathepsin L (CTSL) in the acidic environment of the endosome (15).

Overexpression (OE) screens and loss-of-function (LOF) assays are both powerful tools for identifying and characterizing the roles of interferon-stimulated genes (ISGs) in antiviral immunity. OE assays are effective for identifying ISGs that can act autonomously to inhibit virus replication when overexpressed. However, forced overexpression of genes may result in phenotypes that do not accurately reflect the physiological states. LOF assays, on the other hand, are more likely to identify ISGs that are physiologically relevant. Here, we performed a screen based on 109 ISG-knockout cell lines to pinpoint host factors that inhibit SARS-CoV-2 infection, and PLSCR1 was identified as a key antiviral factor among ISGs.

PLSCR1 is a calcium-dependent type II single-pass transmembrane protein. However, it is not only distributed in membranous structures such as cytoplasmic membrane, Golgi apparatus, and endoplasmic reticulum but also found in the cytoplasm and nucleus (16). This diverse localization pattern enables PLSCR1 to be involved in various cellular processes, including apoptosis, autophagy, cell signaling, membrane dynamics, and so on (17–21). It has been shown to inhibit the replication of several viruses, including hepatitis B virus and hepatitis C virus (22, 23), influenza A virus (24), cytomegalovirus (25), vesicular stomatitis virus and encephalomyocarditis virus (26), and SARS-CoV-2 (12, 13). In our study, we discovered that PLSCR1 suppresses SARS-CoV-2 infection by downregulating cell surface ACE2, uncovering a novel mechanism of PLSCR1’s antiviral action.

## RESULTS

### Identification of ISGs that inhibit SARS-CoV-2 infection by screening ISG-knockout cell lines

Secreted type I IFNs exert antiviral effects by binding to interferon receptors, thereby promoting the expression of a diverse set of ISGs. However, many of the antiviral mechanisms by which ISGs act are still not fully understood. We treated A549 cells with IFNβ and identified a series of ISGs that were stimulated by IFNβ (data not shown). Knockout cell lines were then constructed for these ISGs using the CRISPR-Cas9 technology. Ultimately, we successfully obtained 109 knockout cell lines. ACE2 was subsequently overexpressed in each of these cell lines via lentiviral infection, followed by viral infection assays for screening.

**Fig 1:**
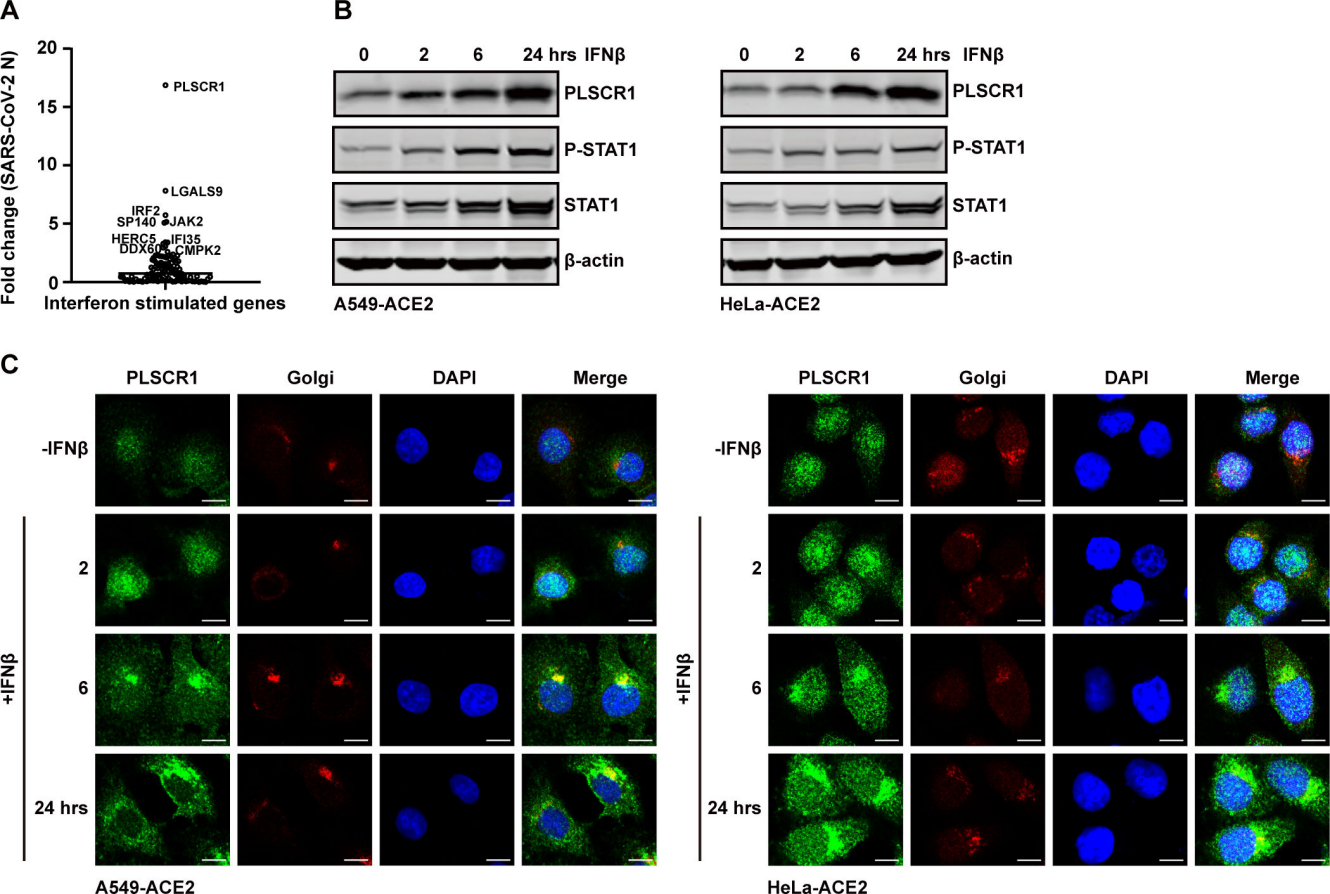
PLSCR1 was identified as a crucial restriction factor against SARS-CoV-2 infection by screening ISG-knockout cell lines, and it can be induced by type I interferon. (**A**) 109 ISG-knockout cell lines were infected with SARS-CoV-2 at an MOI = 0.2 for 24 h. Cells were then harvested, and total RNA was extracted. SARS-CoV-2 RNA levels were quantified using quantitative real-time PCR. (**B**) A549-ACE2 cells and HeLa-ACE2 cells were treated with 500 U/mL of recombinant human IFN-β for 0, 2, 6, and 24 h. PLSCR1 expression, STAT1 level, and the phosphorylation of STAT1 were detected by western blot analyses. (**C**) A549-ACE2 cells and HeLa-ACE2 cells were treated as described in panel (**B**). Cells were fixed and stained with indicated antibodies. Scale bar, 10 µm. All experiments were performed at least twice, and one representative is shown.

These knockout cells were individually infected with SARS-CoV-2 at a multiplicity of infection (MOI) of 0.5, and expression levels of the viral N gene were detected by quantitative RT-PCR (qRT-PCR), which reflected the levels of SARS-CoV-2 replication. We identified several ISGs whose knockout resulted in increased expression of the SARS-CoV-2 N gene, including PLSCR1, LGALS9, IRF2, SP140, HERC5, CMPK2, and host factors associated with interferon signaling pathways (JAK2, DDX60, and IFI35) (Fig. 1A). Among these ISGs, PLSCR1 exhibited most potent inhibitory activity against SARS-CoV-2 replication (Fig. 1A). Additionally, genome-wide association study has predicted that PLSCR1 missense variants was associated with severe COVID-19, suggesting an important role PLSCR1 in antagonizing SARS-CoV-2 infection (12, 13, 27). Therefore, we focus on investigating PLSCR1 in this context. First, we examined whether PLSCR1 expression is inducible by type I IFN. Our results showed that IFN-β treatment induced PLSCR1 expression in a time-dependent manner in A549 and HeLa cells (Fig. 1B). Immunofluorescence assays revealed that PLSCR1 redistributed from the nucleus to the cytoplasm upon IFN-β treatment (Fig. 1C).

### PLSCR1 restricts SARS-CoV-2 infection

**Fig 2:**
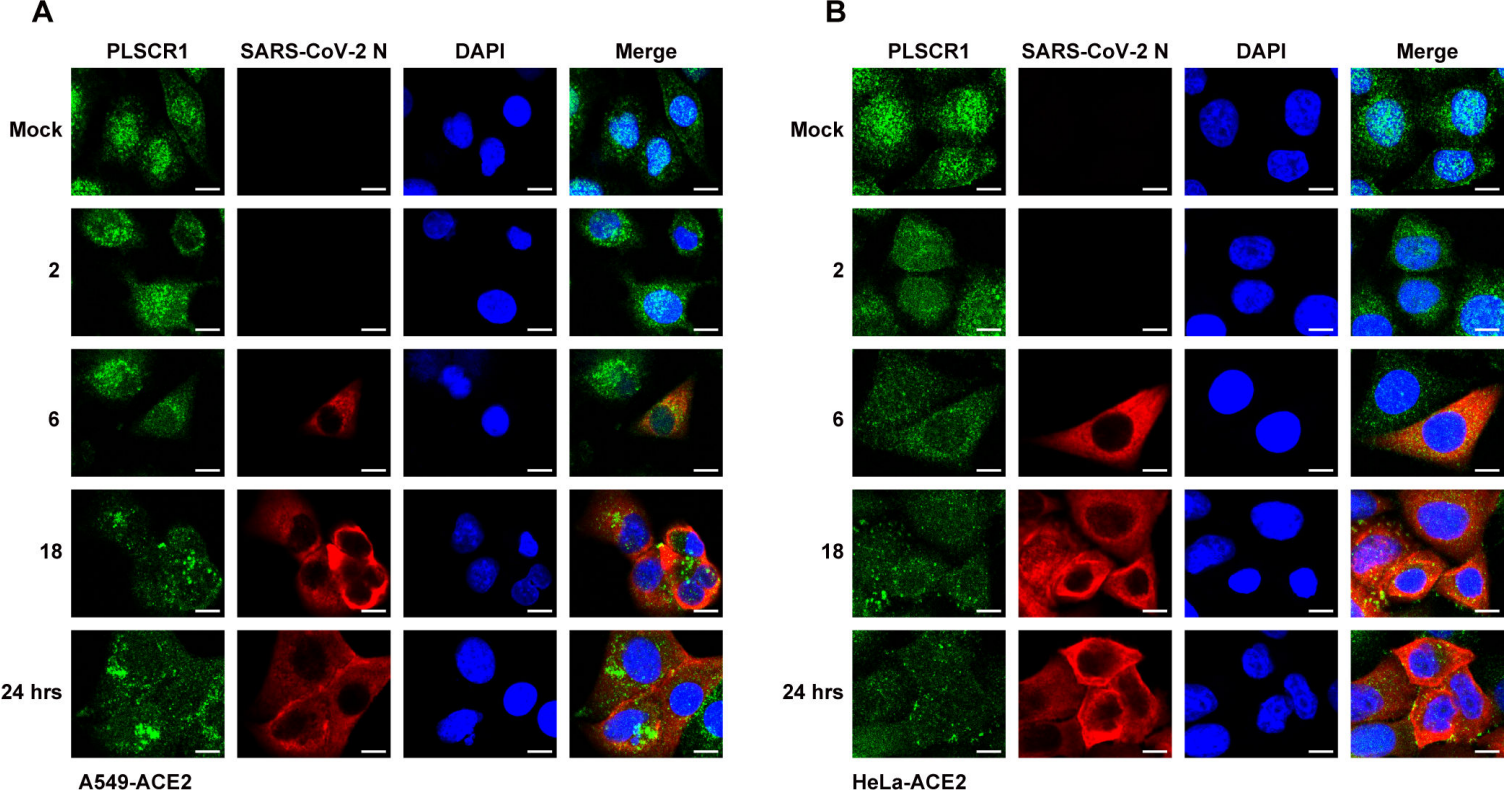
SARS-CoV-2 infection induces the redistribution of PLSCR1. (**A**) A549-ACE2 and (**B**) HeLa-ACE2 cells were infected with SARS-CoV-2 at an MOI = 0.5. At the indicated time points (0, 2, 6, 18, and 24 h post-infection), the cells were fixed and stained to visualize SARS-CoV-2 N (red), PLSCR1 (green), and nuclei (blue). Scale bar, 10 µm. All experiments were performed at least twice; one representative is shown.

**Fig 3:**
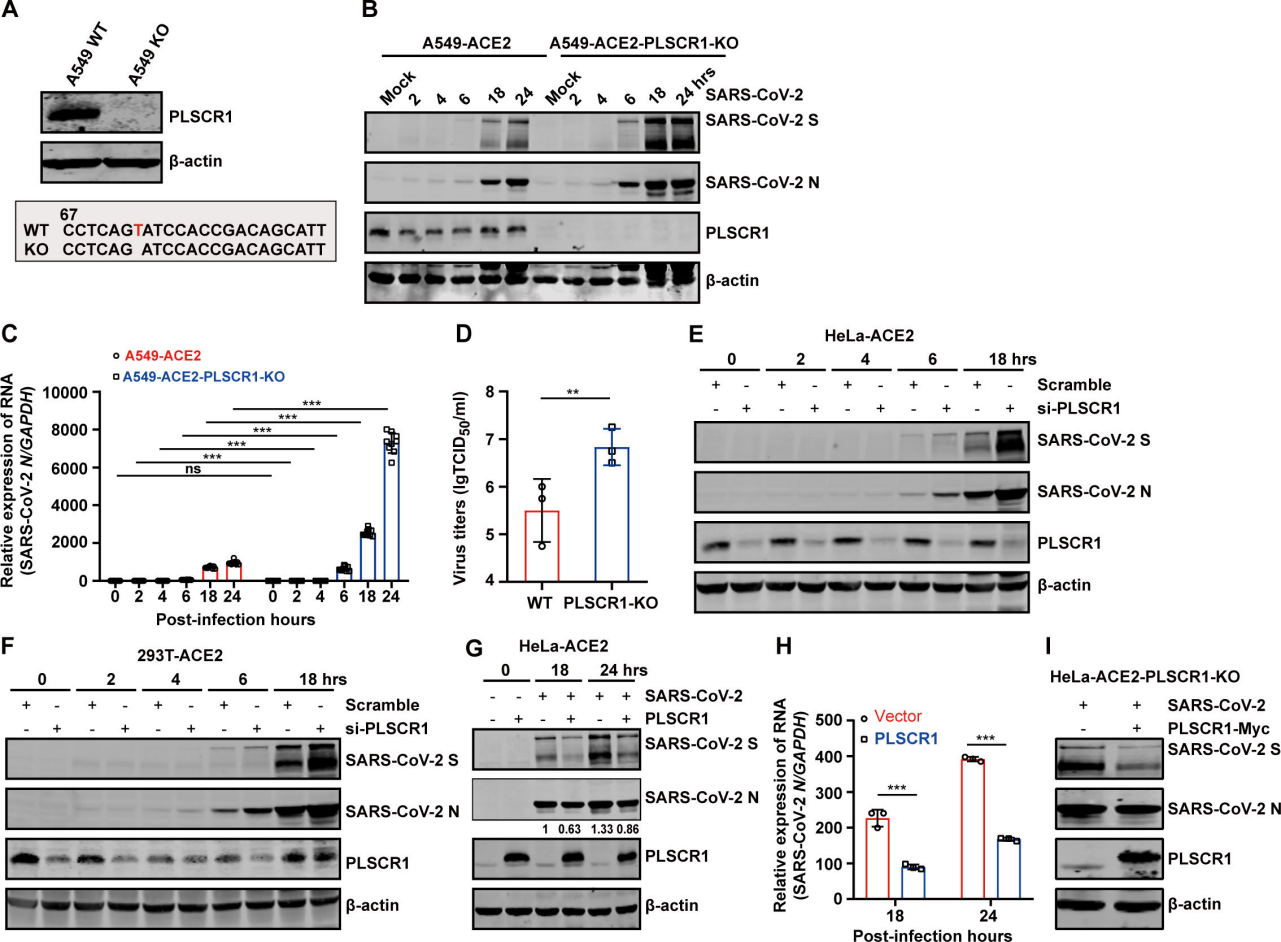
PLSCR1 restricts SARS-CoV-2 infection. (**A**) PLSCR1-KO cells were generated by CRISPR-Cas9 genome editing. The upper panel shows PLSCR1 expression in WT and KO cells. The lower panel displays the sequences of the targeted region. (**B**) A549-ACE2 and A549-ACE2-PLSCR1-KO cells were infected with SARS-CoV-2 at an MOI = 0.5. At the indicated time points (0, 2, 4, 6, 18, and 24 h), cell lysates were collected and analyzed by western blot. (**C**) The cells treated identically to those in panel (**B**) was analyzed by qRT-PCR to measure viral RNA levels. (**D**) A549-ACE2 and A549-ACE2-PLSCR1-KO cells were infected with SARS-CoV-2 at an MOI = 0.5 for 24 h. Supernatants were collected, and the virus titers were determined by TCID_50_ assay. (**E-F**) HeLa-ACE2 and 293T-ACE2 cells were transfected with siRNA targeting PLSCR1. After 48 h, the cells were infected with SARS-CoV-2 at an MOI = 0.5. SARS-CoV-2 replication was assessed by western blot at the indicated time points. (**G**) HeLa-ACE2 and HeLa-ACE2-PLSCR1-OE (overexpressing PLSCR1) cells were infected with SARS-CoV-2 at an MOI = 0.5. Whole-cell lysates were collected at 18 and 24 h post-infection and analyzed by western blot. (**H**) Total RNA extracted from cells treated as in panel (**G**) was extracted and analyzed by qRT-PCR to measure viral RNA levels. (**I**) HeLa-ACE2-PLSCR1-KO cells were transfected with either a vector plasmid or a plasmid expressing PLSCR1. After 24 h of transfection, the cells were infected with SARS-CoV-2 at an MOI = 0.5 for an additional 24 h. Cell lysates were collected and analyzed by western blot. All data are mean value ± SD. These experiments were repeated at least twice. *P* < 0.05 (*), *P* < 0.01(**) and *P* < 0.001(***).

First, we examined the localization of PLSCR1 following SARS-CoV-2 infection. Our results showed that SARS-CoV-2 infection altered PLSCR1 localization in A549-ACE2 cells, causing it to redistribute from being primarily located in the nucleus and plasma membrane to predominantly in the cytoplasm, with a similar effect observed in HeLa-ACE2 cells (Fig. 2A and B). This change in localization mirrors the response seen upon type I interferon treatment, suggesting that PLSCR1 may function as an antiviral factor. To further confirm the role of PLSCR1 in SARS-CoV-2 infection, we generated an independent PLSCR1-knockout cell line using a different sgRNA. PLSCR1 depletion was confirmed by western blot and DNA sequencing analysis, and the cell line was named A549-ACE2-PLSCR1-KO (Fig. 3A). A549-ACE2-PLSCR1-KO and A549-ACE2 cells were, respectively, infected with SARS-CoV-2 (MOI = 0.5). At multiple time points, the cells were harvested to assess the protein expression levels of SARS-CoV-2′s N and S proteins. It was found that depletion of PLSCR1 led to a marked increase in the expression of N and S proteins (Fig. 3B). This upregulation was mirrored at the mRNA level for the N gene in PLSCR1-KO cells (Fig. 3C). Fifty percent tissue culture infectious dose (TCID_50_) assays revealed a significant increase in viral titers in the absence of PLSCR1 (Fig. 3D). Additionally, the role of PLSCR1 in SARS-CoV-2 infection was further confirmed in multiple cell lines, including 293T-ACE2 and HeLa-ACE2, using siRNA-mediated knockdown of PLSCR1 (Fig. 3E and F), suggesting that the PLSCR1’s effect is not cell type-specific.

Next, we investigated whether increasing cellular PLSCR1 levels could modulate SARS-CoV-2 replication. As shown in Fig. 3G, ectopic overexpression of PLSCR1 in HeLa-ACE2 cells significantly inhibited the expression of N and S proteins of SARS-CoV-2. Concurrently, mRNA levels of the N gene were reduced (Fig. 3H). Furthermore, reintroduction of PLSCR1 into PLSCR1-deficient cells inhibited SARS-CoV-2 replication (Fig. 3I). These results collectively indicate that PLSCR1 plays a crucial role in the host’s defense mechanisms against SARS-CoV-2 infection.

### PLSCR1 inhibits cellular entry of SARS-CoV-2

**Fig 4:**
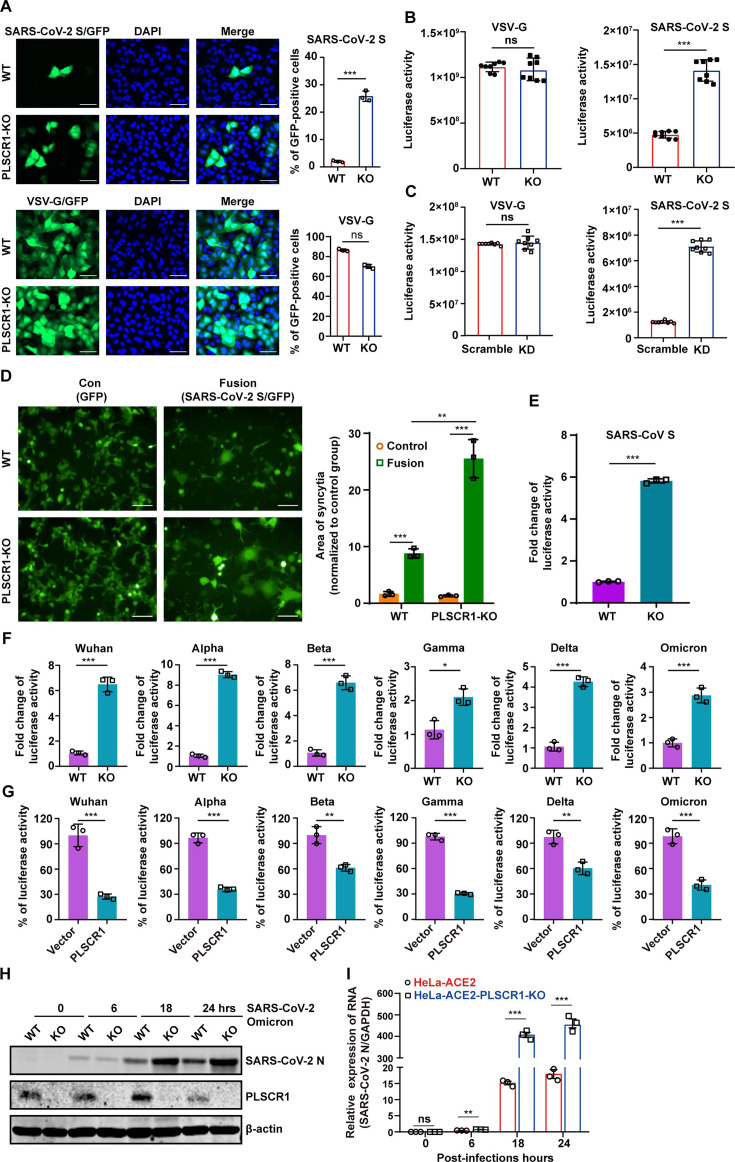
PLSCR1 inhibits SARS-CoV-2 spike pseudovirus infectivity. (**A**) The A549-ACE2 and A549-ACE2-PLSCR1-KO cells were infected with SARS-CoV-2/VSV pseudovirus carrying a GFP reporter gene. Infectivity was indicated by GFP expression, and cell nuclei were stained with DAPI (blue). The histogram on the right displays the percentage of GFP-positive cells. Scale bar, 50 µm. (**B**) Similar to panel (**A**), but the pseudovirus carries a luciferase reporter gene. Infectivity in the A549-ACE2 and A549-ACE2-PLSCR1-KO cells was quantified using a luciferase assay. (**C**) This panel is similar to panel (**B**), except PLSCR1 was knockdown (KD) in A549-ACE2 cells. (**D**) HEK293T cells were transfected with plasmids encoding SARS-CoV-2 S and GFP. After 24 h of transfection, the cells were co-cultured with A549-ACE2 or A549-ACE2-PLSCR1-KO cells for 24 h. Syncytia formation (cell fusion) was observed under a microscope. Scale bar, 100 µm. The histogram on the right displays the area of syncytia, which was measured using Image J. (**E**) A549- ACE2 and A549-ACE2-PLSCR1-KO cells were infected with pseudoviruses-bearing SARS-CoV-S protein. (**F**) A549-ACE2 and A549-ACE2-PLSCR1-KO cells were infected with pseudoviruses bearing S proteins from various SARS-CoV-2 variants, including Wuhan, Alpha, Beta, Gamma, Delta, and Omicron. Infectivity was measured using a luciferase assay. (**G**) Similar to panel (**F**), but HeLa-ACE2 and HeLa-ACE2-PLSCR1-OE cells were used. Infectivity of pseudoviruses was again measured by luciferase assay. (**H**) HeLa-ACE2 and HeLa-ACE2-PLSCR1-KO cells were infected with SARS-CoV-2 Omicron strain at an MOI = 0.5. After 0, 6, 18, and 24 h post-infection, whole cell lysates were collected and subjected to western blot analyses using antibodies specific for PLSCR1, SARS-CoV-2 N protein, and β-actin. (**I**) Total RNA extracted from cells treated as described in (**H**) were analyzed by qRT-PCR. All data are mean value ± SD. These experiments were done in triplicates and repeated at least twice. *P* < 0.05 (*), *P* < 0.01(**) and *P* < 0.001(***).

It has been reported that PLSCR1 is involved in the entry of various viruses (28, 29). To investigate whether PLSCR1 regulates SARS-CoV-2 entry, we constructed SARS-CoV-2 spike pseudotyped viruses (spike-pseudovirus) harboring a GFP reporter gene or a luciferase reporter gene, enabling the assessment of viral entry into host cells. Cells were infected with spike-pseudovirus and subsequently fixed at 48 h post-infection (hpi). The number of GFP-positive cells was significantly elevated in A549-ACE2-PLSCR1-KO cells compared with A549-ACE2 cells (Fig. 4A), indicating that the absence of PLSCR1 promotes SARS-CoV-2 spike-mediated virion entry. Luciferase assays gave rise to similar results when PLSCR1 was either knockout or knockdown (Fig. 4B and C). In the absence of PLSCR1, no significant changes in the number of GFP-positive cells or luciferase activity were observed when cells were infected with vesicular stomatitis virus G glycoprotein (VSV-G) pseudotyped viruses, indicating that PLSCR1’s antiviral effect may be specific to SARS-CoV-2 (Fig. 4A through C). Furthermore, PLSCR1 prevented S- and ACE2-mediated cell-cell fusion, as fusion in the PLSCR1 knockout cells was significantly increased (Fig. 4D).

To verify whether PLSCR1 has broad-spectrum restriction activity, we first tested its ability to inhibit SARS-CoV-S-mediated entry. The results showed that SARS-CoV-S-mediated entry was significantly increased in PLSCR1 knockout cells (Fig. 4E), with a similar magnitude to the inhibitory effect observed on SARS-CoV-2-S-mediated entry (Fig. 4F). Since its initial identification in late 2019, SARS-CoV-2 has undergone numerous mutations, leading to the emergence of different variants. We next investigated whether PLSCR1 could inhibit entry of SARS-CoV-2 variants. Luciferase assays demonstrated that PLSCR1 inhibited entry of multiple dominant SARS-CoV-2 strains, including the original Wuhan strain, and the Alpha, Beta, Gamma, Delta, and Omicron variants (Fig. 4F and G), although the restriction is slightly weaker in omicron (Fig. 4F). Consistent with these findings, PLSCR1 also inhibited replication of the authentic Omicron variant of SARS-CoV-2 (Fig. 4H and I), indicating that PLSCR1 exerts a broad-spectrum inhibitory effect against multiple SARS-CoV-2 variants.

**Fig 5:**
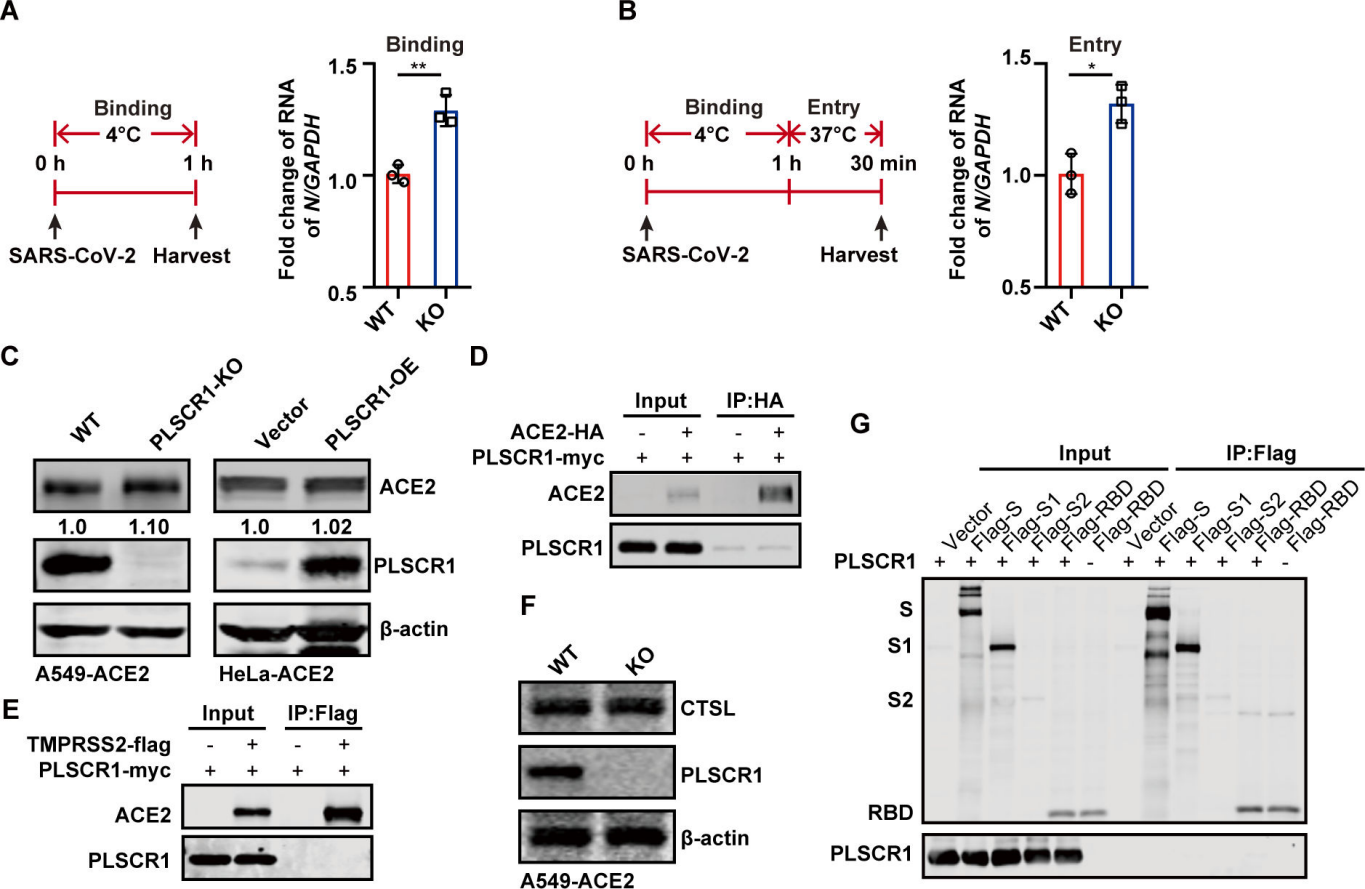
CRISPR/Cas9-mediated PLSCR1 knockout promotes SARS-CoV-2 entry. (**A**) A549-ACE2 and A549-ACE2-PLSCR1-KO cells were infected with SARS-CoV-2 at an MOI = 5. Cells were incubated on ice for 1 h to allow virus binding without internalization. Cells were then harvested and subjected to qRT-PCR to measure SARS-CoV-2 N mRNA levels. (**B**) Cells were treated as in panel (**A**) and were then incubated at 37°C for 30 min to allow viral internalization. Cells were harvested and subjected to qRT-PCR analysis. These experiments were done in triplicates and repeated at least two twice. *P* < 0.05 (*), *P* < 0.01(**), and *P* < 0.001(***). (**C**) Western blot analysis ACE2 expression levels in A549-ACE2, A549-ACE2-PLSCR1-KO, and HeLa-ACE2-Vector/PLSCR1-OE cells. (**D**) Co-IP assay was performed in 293T cells co-expressing HA-tagged ACE2 and myc-tagged PLSCR1. (**E**) Co-IP assay was performed in 293T cells co-expression flag-tagged TMPRSS2 and myc-tagged PLSCR1. (**F**) Western blot analysis of CTSL expression in A549-ACE2 and A549-ACE2-PLSCR1-KO cells. (**G**) Co-IP assay performed in 293T cells expressing various forms of SARS-CoV-2 S protein (full-length S, S1, S2 subunits, or RBD) along with myc-tagged PLSCR1. These experiments were repeated at least twice.

Subsequently, we evaluated the effect of PLSCR1 on SARS-CoV-2 binding and entry using the authentic virus. Upon infection, the cells were incubated with the virus at 4°C for 1 h to allow viral attachment, followed by shifting the temperature to 37°C for an additional 30 min to facilitate viral entry. By measuring the level of N gene through qRT-PCR, it was found that the early viral entry was significantly enhanced in the absence of PLSCR1 (Fig. 5A and B). These findings collectively demonstrated that PLSCR1 functions as a critical restriction factor that impedes SARS-CoV-2 entry.

### PLSCR1 does not directly interact with ACE2 and S protein nor does it rely on its scramblase activity to inhibit SARS-CoV-2

To determine the molecular mechanisms by which PLSCR1 inhibits SARS-CoV-2 entry, we first evaluated the expression of ACE2, the primary receptor of SARS-CoV-2, in cells with PLSCR1 knockout or overexpression. The total amount of ACE2 protein in A549-ACE2 and HeLa-ACE2 cells was not significantly affected by either PLSCR1 knockout or overexpression (Fig. 5C). Furthermore, co-immunoprecipitation experiments showed that PLSCR1 does not directly interact with ACE2 or TMPRSS2, a critical protease for SARS-CoV-2 plasma membrane fusion (Fig. 5D and E). Likewise, PLSCR1 did not alter the expression of CTSL, a protease involved in the endosomal fusion of the virus (Fig. 5F). Additionally, PLSCR1 failed to interact with viral S protein or its subunits (S1, S2, and RBD) (Fig. 5G). These results suggest that the inhibitory effect of PLSCR1 on SARS-CoV-2 entry does not occur through direct interactions with either the host cell receptors or the viral S protein.

**Fig 6:**
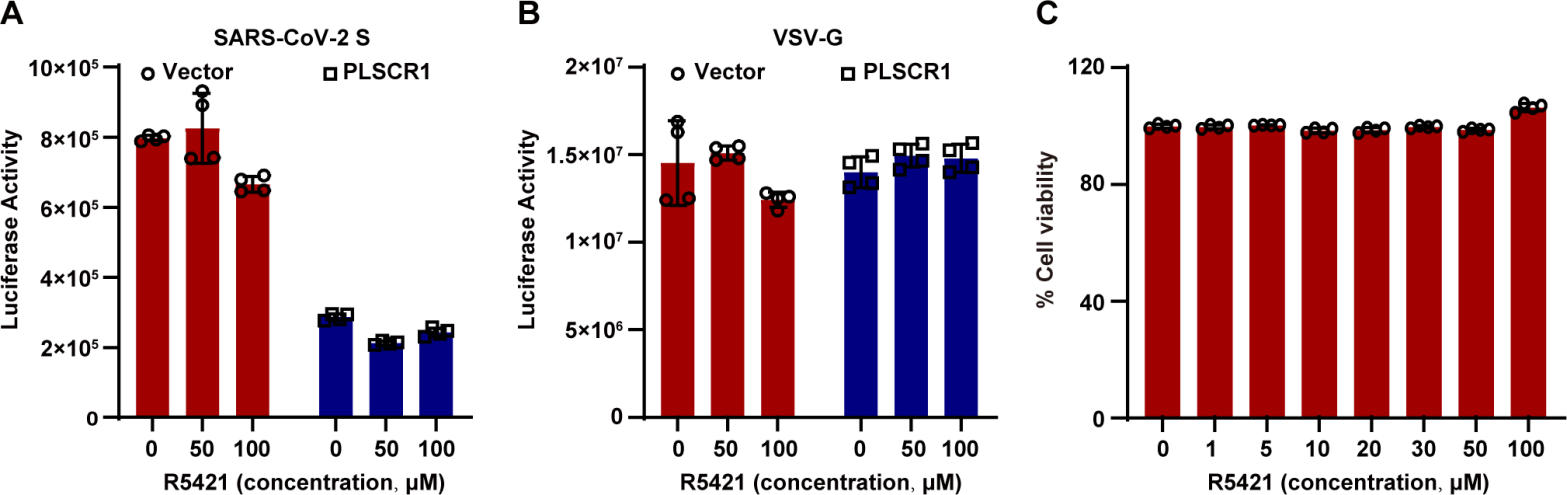
PLSCR1 inhibits SARS-CoV-2 entry independently of its scramblase activity and interferon signaling. (**A**) HeLa-ACE2-PLSCR1-KO cells were transfected with either an empty plasmid or with a plasmid expressing PLSCR1. After 4 h, cells were treated with different concentrations of R5421. After 24 h of transfection, cells were infected SARS-CoV-2 spike pseudovirus for 48 h. Viral infectivity was measured via a luciferase reporter assay. (**B**) This panel is similar to panel (**A**), except cells were infected with VSV-G pseudovirus. (**C**) HeLa-ACE2-PLSCR1-KO cells were treated with increasing concentrations of R5421 for 48 h. Cell viability was assessed by a CCK8 assay. These experiments were done in triplicates and repeated at least two times.

We then examined R5421 (28), a pharmacological inhibitor of PLSCR1’s scramblase activity, for its efficacy on SARS-CoV-2 infection. As shown in Fig. 6A, R5421 treatment did not affect spike-pseudovirus infectivity, regardless of whether PLSCR1 was overexpressed (Fig. 6A). The concentrations of R5421 used in the experiment were non-toxic to cells and did not impact VSV-G pseudovirus infection (Fig. 6B and C). These findings suggest that PLSCR1 likely inhibits viral entry through a mechanism independent of its scramblase activity.

### PLSCR1 inhibits the localization of ACE2 on the plasma membrane

**Fig 7:**
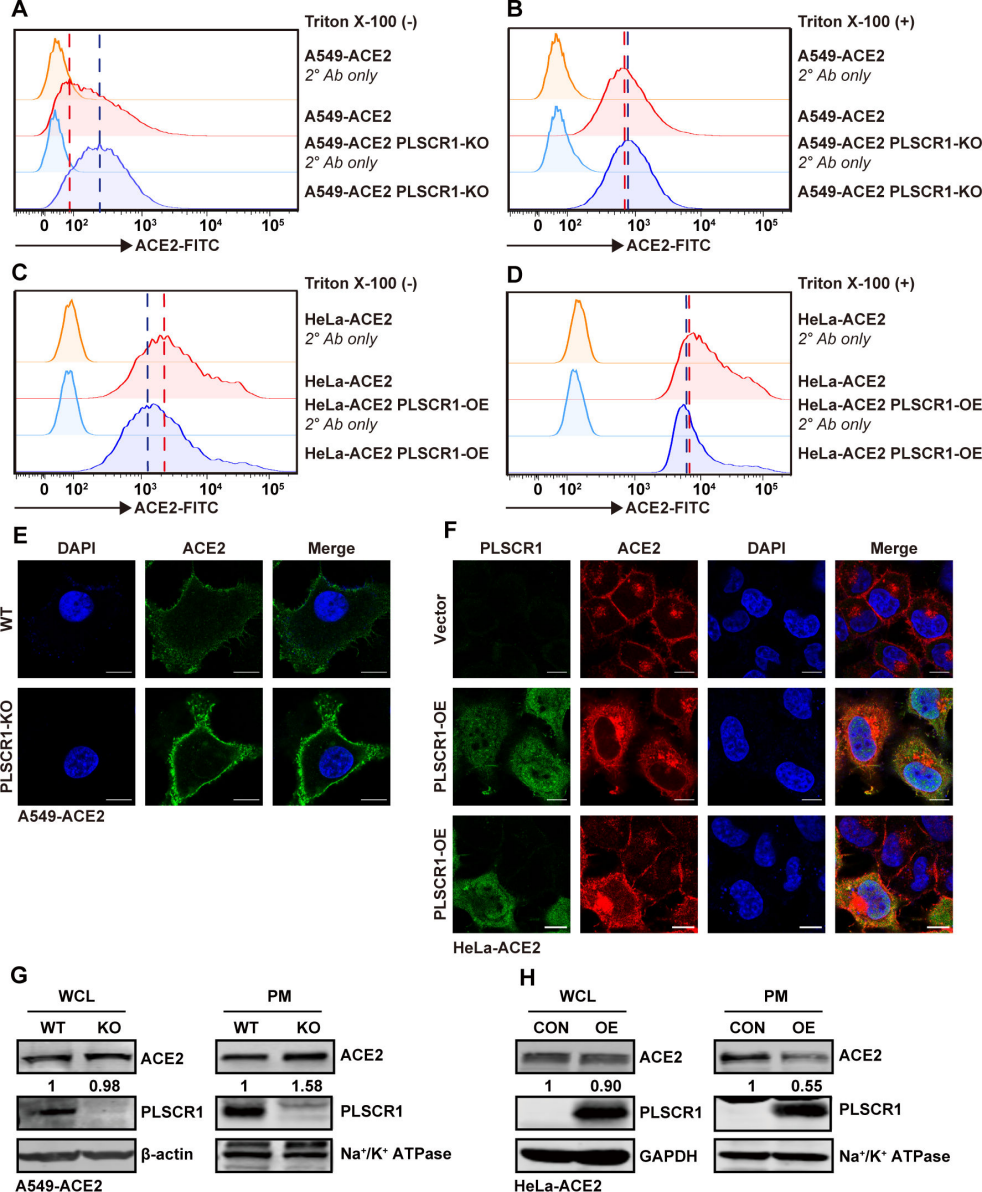
PLSCR1 inhibits the surface distribution of ACE2 on the plasma membrane. (**A-B**) Flow cytometry (FACS) was used to analyze ACE2 expression in A549-ACE2 and A549-ACE2-PLSCR1-KO cells. Panel (**A**) shows the surface expression of ACE2, whereas panel (**B**) shows the total cellular expression of ACE2. (**C-D**) HeLa-ACE2-PLSCR1-KO cells were transfected with either an empty vector or a plasmid expressing PLSCR1 for 24 h. FACS plots display surface ACE2 expression (**C**) and total ACE2 expression (**D**). (**E**) A549-ACE2 and A549-ACE2-PLSCR1-KO cells were fixed and stained to visualize ACE2 (green) and nuclei (blue). Scale bar, 10 µm. (**F**) HeLa-ACE2-PLSCR1-KO cells were transfected with either an empty vector or a plasmid expressing PLSCR1 for 24 h, then fixed and stained to visualize ACE2 (red), PLSCR1 (green), and nuclei (blue). Scale bar, 10 µm. (**G**) A549-ACE2 and A549-ACE2-PLSCR1-KO cells were subjected to plasma membrane (PM) fractionation. Whole cell lysates and PM fractions were analyzed by western blot. Na^+^/K^+^-ATPase was used as a plasma-specific marker. (**H**) HeLa-ACE2-PLSCR1-KO cells were transfected with an empty vector or a plasmid expressing PLSCR1 for 24 h. PMs were isolated and analyzed similarly to panel (**G**). These experiments were repeated at least three times.

SARS-CoV-2 binds to ACE2 to enter host cells, primarily through two mechanisms: direct fusion with the plasma membrane or via endocytosis followed by fusion with the endosomal membrane. In both processes, binding of ACE2 by SARS-CoV-2 on the plasma membrane is pivotal for viral entry. Although PLSCR1 does not affect the overall levels of ACE2 protein (Fig. 5C), its impact on the localization of ACE2 at the plasma membrane needs investigation. We performed immunostaining using specific antibodies against ACE2 and conducted flow cytometry analysis under membrane-impermeable conditions to specifically detect ACE2 present on the plasma membrane, and under membrane-permeable conditions to measure the total ACE2 levels throughout the cells. The results showed that the intensity of ACE2 on the plasma membrane was higher in PLSCR1 knockout cells compared with wild-type cells (Fig. 7A), whereas the intensity of total ACE2 remained unchanged (Fig. 7B). Conversely, plasma membrane ACE2 was reduced upon PLSCR1 overexpression (Fig. 7C), with total ACE2 levels remaining unaltered (Fig. 7D). Immunofluorescence assays confirmed that PLSCR1 deficiency increased plasma membrane ACE2 levels (Fig. 7E), whereas PLSCR1 overexpression decreased plasma membrane ACE2 levels (Fig. 7F). This observation was further substantiated by a plasma membrane isolation assay, which demonstrated that PLSCR1 depletion resulted in elevated ACE2 levels in the plasma membrane fraction (Fig. 7G), whereas overexpression of PLSCR1 had the opposite effect (Fig. 7H). In each of these scenarios, PLSCR1 did not affect total ACE2 levels (Fig. 7G and H). Together, these results suggest that PLSCR1 specifically modulates the localization of ACE2 to the plasma membrane without altering its overall expression within the cell. Since plasma membrane-resident ACE2 is critical for SARS-CoV-2 binding and entry, it can be concluded that PLSCR1 exerts its antiviral function via downregulating ACE2 on the cell surface.

### Impact of the H262Y mutation on PLSCR1 activity

**Fig 8:**
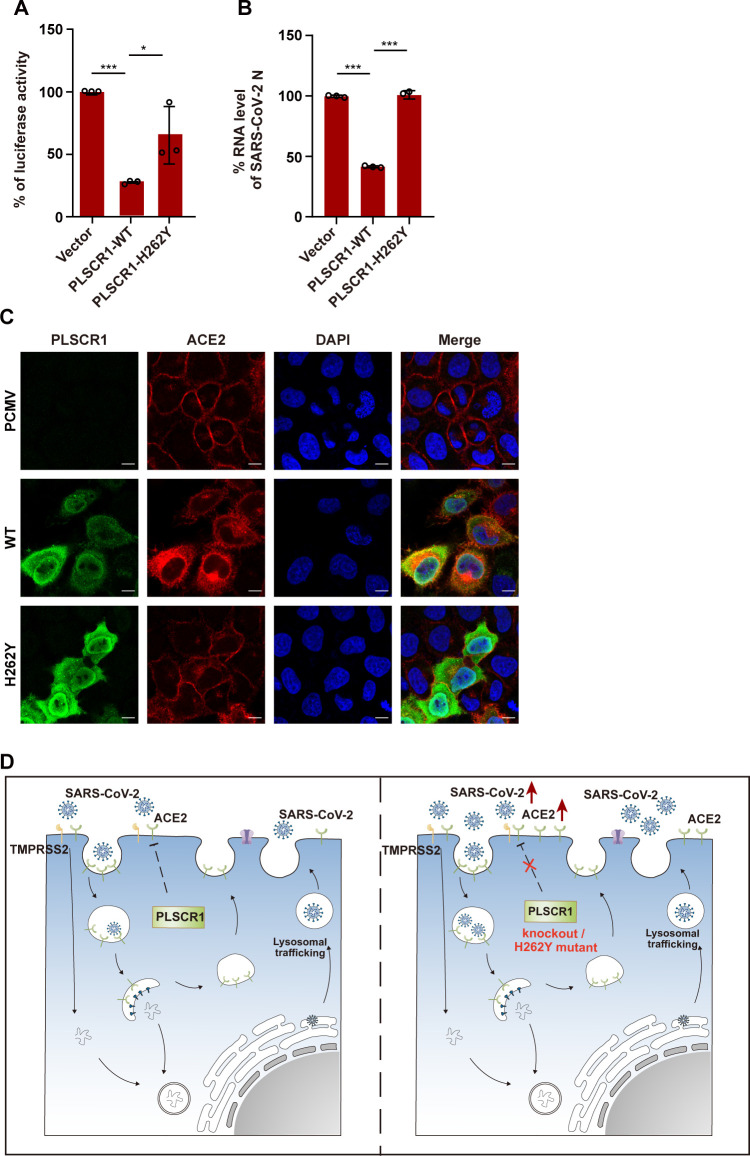
Effect of the absence of PLSCR1 functional site on SARS-CoV-2. (**A-B**) The HeLa-ACE2 cells were transfected with an empty vector, plasmids expressing PLSCR1 of WT or H262Y mutant for 24 h. Cells were then infected with pseudovirus (**A**) for 48 h or SARS-CoV-2 (**B**) for 24 h. Viral infectivity was measured using a luciferase assay (**A**). Total RNA was extracted, and viral RNA levels were measured by qRT-PCR (**B**). (**C**) Empty vector, PLSCR1 wild-type and H262Y mutant were transfected into HeLa-ACE2-PLSCR1-KO cell line for 24 h. Cells were fixed and stained to visualize ACE2 (red), PLSCR1 (green), and nuclei (blue). Scale bar, 10 µm. These experiments were repeated at least three times. (**D**) Schematic diagram illustrating the proposed mechanism by which PLSCR1 antagonizes SARS-CoV-2 infection.

The H262Y mutation in PLSCR1 has been linked to increased susceptibility to severe COVID-19, as previously suggested by whole-genome sequencing data (13, 27). Additionally, recent studies have indicated that this mutation impairs the antiviral function of PLSCR1 (12, 13), although the precise mechanisms by which it disrupts these functions remain unclear. To further investigate the role and mechanism of H262Y mutation, we expressed H262Y mutant in HeLa-ACE2-PLSCR1-KO cells. Using both pseudotyped and authentic SARS-CoV-2 virions, we found that the H262Y mutation in PLSCR1 partially abrogated its inhibitory effect of PLSCR1 on virus entry (Fig. 8A and B). We also examined whether the H262Y mutation affects ACE2 localization. Immunofluorescence assays revealed that overexpression of wild-type PLSCR1 led to reduced plasma membrane ACE2, whereas the H262Y mutant failed to do so, indicating a loss of function in the mutant protein (Fig. 8C). Thus, the H262Y mutation in PLSCR1 impairs its capacity to downregulate cell surface ACE2, compromising its antiviral activity against SARS-CoV-2. Overall, these results propose that PLSCR1 employs an antiviral strategy, potentially targeting host factors and altering its localization to suppress SARS-CoV-2 infection as summarized in Fig. 8D.

## DISCUSSION

ISGs are known for their diverse mechanisms in combating viral infections. Here, we screened 109 ISG-knockout cell lines to identify ISGs that restrict SARS-CoV-2 infection. The screen results encompassed a range of candidate ISGs that have been previously identified for their antiviral activities against SARS-CoV-2. For instance, HERC5 could enhance the innate immune response by promoting ISGylation of various targets to promote interferon production, thereby boosting the innate immune response against SARS-CoV-2 (30–33). Galectin-9, encoded by LGALS9, may inhibit SARS-CoV-2 infection by blocking viral entry or modulating the host immune response (34–36). Additionally, CMPK2, which is upregulated in COVID-19 patients’ leukocytes, may play key roles in antiviral immunity and has been positioned as a therapeutic target against SARS-CoV-2 and other RNA viruses (37, 38). These findings suggest that our strategy is effective for identifying ISGs that antagonize SARS-CoV-2 replication.

Among the identified ISGs, PLSCR1 stood out as a key antiviral factor, with its knockout having the most pronounced impact on viral replication. This underscores its significant role in the host-SARS-CoV-2 interaction. As an ISG, PLSCR1 demonstrated potential antiviral effects by suppressing the production of the viral N and S proteins and by markedly reducing virus titers. Pseudovirus assays further demonstrated that PLSCR1 effectively blocks SARS-CoV-2 entry. These findings align with previous studies showing that PLSCR1 inhibits SARS-CoV-2 S protein-mediated fusion, thereby preventing viral entry (12). Notably, PLSCR1 was shown to impede the entry of multiple SARS-CoV-2 variants, including Alpha, Beta, Gamma, Delta, and Omicron, underscoring its potential as a broad-spectrum therapeutic target for COVID-19 and future coronavirus outbreaks. Recent work by Le Pen et al. linked PLSCR1 to COVID-19 outcomes and emphasized its antiviral effects through functional screens. Their findings also revealed that PLSCR1 suppresses infections caused by various SARS-CoV-2 variants, although its inhibitory activity is somewhat diminished against newly emerging variants (13). This observation underscores the importance of further investigating PLSCR1’s effect on future variants.

ACE2, serving as the primary receptor for SARS-CoV-2, is predominantly expressed on the surface of various human cells and exhibits dynamic behavior characterized by continuous internalization and recycling processes (39). Rab7a, a small GTPase protein, plays a role in vesicular trafficking and has been shown to regulate cell surface localization of ACE2. Rab7a deletion resulted in ACE2 sequestration within endosomal vesicles (14, 39). Our observations suggested that PLSCR1 decreased ACE2 levels on the plasma membrane without affecting total ACE2 levels. Although the flow cytometry assay results from Pen et al. did not show a significant change in ACE2 expression on the cell membrane surface (13), our immunofluorescence data more directly illustrated the impact of PLSCR1 on the distribution of ACE2 at the membrane surface. Additionally, the H262Y mutation in PLSCR1 impairs its capacity to downregulate cell surface ACE2, compromising its antiviral activity against SARS-CoV-2. The H262Y mutation is located within the nuclear localization sequence of PLSCR1, but similar to the wild-type protein, the H262Y mutant is distributed both in the nucleus and the cytoplasm. Therefore, the H262Y mutation likely alters the function of PLSCR1 through a mechanism that does not involve changes to its nuclear localization. Further investigation is needed to elucidate the mechanism by which the site affects antiviral activity of PLSCR1.

However, the precise mechanisms by which PLSCR1 downregulates surface ACE2 localization remain to be fully elucidated. Several potential mechanisms could underlie the effects of PLSCR1: (i) PLSCR1 may modulate the intracellular transport of nascent ACE2, affecting its plasma membrane presence; (ii) PLSCR1 may regulate endocytosis or recycling of ACE2; (iii) interactions with trafficking or membrane dynamic proteins like Rab7a might be involved; and (iv) PLSCR1 may impact membrane lipid organization. Of note, R5421, an inhibitor of PLSCR1 scramblase activity, does not effectively inhibit SARS-CoV-2 entry, suggesting that PLSCR1’s role in restricting viral entry may be independent of its enzymatic scramblase function. This observation is consistent with the report by Xu D. et al., who showed that the scramblase activity of PLSCR1 is relatively weak than that of other lipid scramblases, such as TMEM16F (12), and that this scramblase activity is not required for PLSCR1 to restrict SARS-CoV-2. Therefore, other factors or mechanisms might be involved in PLSCR1’s anti-SARS-CoV-2 effects, necessitating further investigation.

Xu et al. suggest that PLSCR1 directly targets SARS-CoV-2-containing vesicles to prevent spike-mediated fusion and viral escape (12). Our study revealed that PLSCR1 exerts its functions by reducing ACE2 on the plasma membrane. Despite the variety of mechanisms proposed, the collective research findings consistently demonstrate PLSCR1’s inhibitory effect on SARS-CoV-2 entry. These findings highlight the multifaceted nature of host-virus interactions, and the diverse strategies employed by host cells to counteract viral infection.

Taken together, our study highlights the importance of investigating the role of ISGs in the context of innate immune response to SARS-CoV-2 infection. A thorough investigation into the function and mechanism of action of PLSCR1 will help further understand the mechanism of host antiviral strategies. The detailed mechanism by PLSCR1 regulates the trafficking and recycling of ACE2 awaits further investigation.

## MATERIALS AND METHODS

### Cell lines and virus

HEK293T (ATCC, #CCL-11268), HeLa (ATCC, #CCL-2), Huh7.5.1, HeLa-ACE2, A549-ACE2, A549-ACE2-PLSCR1-KO, and HeLa-ACE2-PLSCR1-KO cells were cultured in Dulbecco’s modified Eagle’s medium (DMEM) supplemented with 10% (vol/vol) heat-inactivated fetal bovine serum (FBS) and 1% penicillin-streptomycin at 37 ℃ in a 5% CO_2_ incubator. HeLa-ACE2 and A549-ACE2 cell lines were established by stably transfected with a plasmid expressing ACE2 as previously described (40). A549-ACE2-PLSCR1-KO cells and HeLa-ACE2-PLSCR1-KO cells were generated through Cas9-mediated editing. The PLSCR1-sgRNA sequences used were “CCTCAGTATCCACCGACAGCATT” and “CGGAAACAAACTTGCCAGTT.” The SARS-CoV-2 virus was isolated from respiratory samples of COVID-19 patients in our lab and propagated in Vero cells for use in this study (41). All experiments involving the SARS-CoV-2 virus were conducted in the BSL-3 laboratory.

### Antibodies

Primary antibodies were as follows: rabbit anti-PLSCR1 mAb from Sigma, (Cat# HPA068923); mouse anti-SARS-CoV/SARS-CoV-2 Nucleocapsid mAb from Sino Biological (Cat# 40143-MM08); rabbit anti-SARS-CoV-2 (2019-nCoV) Spike pAb from Sino Biological (Cat# 40591-T62); rabbit anti-ACE2 pAb from Abclonal (Cat# A12737); mouse anti-Flag mAb from Sigma (Cat# F3165); mouse anti-β-actin mAb from Sigma (Cat# A5441); mouse anti-HA mAb from Sigma (Cat# H9658); and rabbit anti-Na^+^/K^+^-ATPase mAb from Invent (Cat# IN-SM005AB).

### Plasmid construction

The following plasmids were utilized and stored at the Christophe Merieux Laboratory: Myc-PLSCR1, pCMV6-entry, eGFP-C1, ACE2, pLenti-CRISPRv2, pCAGGS-S (SARS-CoV-2 Wuhan, Alpha, Beta, Gamma, Delta, and Omicron), psPAX2, pVSVG, pLenti-GFP, pLenti-GFP/luc, pMDIG, RSV-REV, and pCDH-CMV-MCS-EF1-Puro. The H262Y mutant of the PLSCR1 was constructed using a Site-Directed Mutagenesis Kit (Stratagene, La Jolla, CA) according to the manufacturer’s instructions and was verified by sequencing. The primers used for the H262Y mutant were Forward: 5′-GTGTGTGGTTGGCAAAATTTCCAAGT-ACTGGACTGGAATTTTGA-3′ and Reverse: 5′-GCCTCTCTCAAAATTCCAGTCCA-GTACTTGGAAATTTTGCCAAC-3′.

### Western blot

Cells were lysed in a buffer containing 150 mM NaCl, 25 mM Tris (pH 7.4), 1% NP40, 0.25% sodium deoxycholate, and 1 mM EDTA, supplemented with a protease inhibitor cocktail. Protein lysates were then mixed with loading buffer and boiled to denature the proteins. The samples were subjected to SDS-PAGE for separation based on molecular weight and subsequently transferred onto nitrocellulose membranes. Membranes were blocked with 5% skim milk, followed by incubation with primary antibodies for 3 h at room temperature or overnight at 4°C. After washing, membranes were incubated with IRDye Fluor 680/800-labeled secondary antibodies for 30 min away from light. Following additional washes, the membranes were scanned using the Odyssey infrared imaging system at wavelengths of 700 and 800. Protein sizes were estimated by comparison with pre-stained molecular weight markers (Elementas, Rockville, MD).

### Real-time PCR

Total RNA was extracted using the TRIzol reagent (Invitrogen) and reverse-transcribed using M-MLV Reverse Transcriptase (Promega) according to the manufacturer’s instructions. Real-time quantitative PCR was conducted using TB Green Premix Ex Taq (Tli RNaseH Plus) (Takara Bio). The following primers were used for qPCR: PLSCR1-Forward: 5′-AGAACAGCTTTGGACAGAGGGTTTACTTTGC-3′, PLSC-R1-Reverse: 5′-CTGAAGGCAGCAGGGACAACAACAG-3′, GAPDH-Forward: 5′-CAACTGGTCGTGGACAACCAT-3′, GAPDH-Reverse: 5′-GCACGGACACTCACAATGTTC-3′, CTSL-Forward: 5′-AAACTGGGAGGCTTATCTCACT-3′, and CTSL-Reverse: 5′-GCATAATCCATTAGGCCACCAT-3′. Primer sequences specific to the SARS-CoV-2 were available upon request from J.W.

### Fifty percent tissue culture infectious dose (TCID_50_) assays

Samples were subjected to three freeze-thaw cycles, followed by low-speed centrifugation to remove cellular debris. Vero cells were seeded overnight in 96-well plates to research approximately 80% confluency. Serial 10-fold dilutions of the samples were prepared; 100 µL/well of each dilution were added to the Vero cells in octuplicate. Cells were incubated at 37°C with 5% CO_2_ for 1 h. The culture medium was then replaced with opti-MEM containing 1% BSA, and the cells were incubated for an additional 4 days to allow for cytopathic effect (CPE) development. The CPE was assessed under a microscope and recorded.

### Co-immunoprecipitation

In total, 3 × 10^6^ cells were seeded in 6-well plates. After transfection with the indicated plasmids for 24 h, the cells were harvested and lysed in radioimmunoprecipitation assay (RIPA) buffer (150  mM NaCl, 1% NP40, 20  mM Tris-HCl, 1 mM EDTA, pH 7.4, and 0.25% sodium deoxycholate) supplemented with complete protease inhibitors (Roche) for 30  min. The lysates were centrifuged at 12,000 × *g* for 10 min at 4°C. A 60 µL aliquot of the supernatant was saved as “Input,” whereas the remainder was incubated with Flag antibody overnight at 4°C. The following day, Protein A/G plus agarose beads were washed and added to the lysates. Following 2-h incubation at 4°C, the beads-lysate complexes were centrifuged and thoroughly washed to eliminate non-specific interactions. Afterward, a loading buffer was added to the samples, which were then boiled to denature the proteins and detach them from the beads. Samples were analyzed by western blot.

### Immunofluorescence assay

Cells were washed with PBS and fixed in 4% paraformaldehyde (BOSTER) for 10–20 min at room temperature. Cells were then permeabilized with 0.5% Triton-X-100 and washed again with PBS. Following this, the cells were blocked with 5% BSA. Primary antibodies were added, and the cells were incubated overnight at 4°C. The next day, secondary antibodies and 4′,6-diamidino-2-phenylindole (DAPI) (for nuclear staining) were added and incubated for 1 h at room temperature. Protein localization was visualized using laser confocal microscopy.

### Binding and entry of virus

Cells were seeded into confocal dishes and culture for 24 h. Afterward, cells were infected with SARS-CoV-2 at an MOI = 5. To perform the virus binding assay, cells were placed on ice for 1 h to allow virus attachment. Following this, cells were washed twice with cold PBS to remove unbound virions that did not interact with the cell receptor. Whereafter, cells were incubated at 37°C for 30 min to allow for viral entry. Cells were washed twice more with cold PBS and harvested for further analysis.

### Construction of SARS-CoV-2-spike pseudovirus system

HEK293T cells were transfected with the plasmids pCAGGS-S (SARS-CoV-2 S) or VSV-G, along with psPAX2 and plenti-GFP/luciferase. After 48  h of transfection, the supernatant was collected in a 50 mL centrifuge tube and centrifuged at 2,600 g for 10 min to remove debris. The clarified supernatant was aliquoted and stored at −80 ℃ for late use. Pseudovirus infection was confirmed by detecting GFP expression or luciferase activity. After infection, cells were lysed with 40 µL 1× passive lysis buffer (PLB), and luciferase activity was measured according to the Bright-Lite Luciferase Assay System protocol (Vazyme, Cat# DD1204). Relative luminescence units (RLU) were determined using EnSight Multimode Plate Reader (PerkinElmer, Cat# HH3400). GFP distribution in infected cells was observed and documented using fluorescence microscopes.

### Cell fusion assay

HEK293T cells were transfected with plasmids encoding SARS-CoV-2 S protein and GFP. After 24 h of transfection, these HEK293T cells (donor cells) overexpressing SARS-CoV-2 S/GFP were mixed with A549-ACE2 or A549-ACE2-PLSCR1-KO cells (acceptor cells) in a 1:3 specific ratio. The mixed cells were then co-cultured for 24 h. After co-culturing, cells were washed with PBS, fixed by 4% PFA, and visualized using fluorescence microscopes.

### Flow cytometry

Cells were harvested and washed with 1× PBS, after which all subsequent steps were performed on ice. Then, cells were either permeabilized or non-permeabilized, dependent on the experiment requirement. Cells were then incubated with a primary anti-ACE2 antibody for 20 min away from light. After incubation, 1 mL of LB reagent was added to the cells to stop the reaction. The cells were centrifuged at 300 g for 5 min, and the supernatant was discarded. Next, the secondary antibody Rb488 was added, and the cells were incubated for 15 min. The reaction was terminated by adding 1 mL of LB reagent. Following a second round of centrifugation and removal of the supernatant, the cells were suspended in 200 µL of PBS and analyzed by flow cytometry. Flow cytometry data were processed using FlowJo version 7.6.

### Plasma membrane fractionation

Plasma membranes (PMs) were isolated by using a cell fractionation kit (Invent Biotechnologies, SM-005) according to the manufacturer’s instructions.

### Statistical analysis

All data are presented as mean ± standard deviation (SD). Statistical analysis was performed using independent samples *t*-test with Graph Prism software. Significance levels are indicated as follows: *P  < 0.05, **P  < 0.01, ***P  < 0.001.
